# Supplementary material for: Multi‐dataset identification of innovative feature genes and molecular mechanisms in keratoconus
Source: J Cell Mol Med. 2024 Sep 19;28(18):e70079. doi: 10.1111/jcmm.70079 (PMC11412914; doi:10.1111/jcmm.70079)
Supplement: Supplementary file 2 — Material S2. [file JCMM-28-e70079-s002.zip › 7.GSEA analysis/1.ARL11/KEGG_TGF_BETA_SIGNALING_PATHWAY.html]

Details for gene set KEGG\_TGF\_BETA\_SIGNALING\_PATHWAY[GSEA]

|  || Dataset | ARL11.group.cls#h\_versus\_l |
| Phenotype | group.cls#h\_versus\_l |
| Upregulated in class | l |
| GeneSet | KEGG\_TGF\_BETA\_SIGNALING\_PATHWAY |
| Enrichment Score (ES) | -0.3534988 |
| Normalized Enrichment Score (NES) | -1.1713991 |
| Nominal p-value | 0.26757812 |
| FDR q-value | 1.0 |
| FWER p-Value | 0.989 |
Table: GSEA Results Summary

  

Fig 1: Enrichment plot: KEGG\_TGF\_BETA\_SIGNALING\_PATHWAY      
 Profile of the Running ES Score & Positions of GeneSet Members on the Rank Ordered List

  

| SYMBOL | TITLE | RANK IN GENE LIST | RANK METRIC SCORE | RUNNING ES | CORE ENRICHMENT || 1 | TGFBR2 | na | 2250 | 0.279 | -0.0328 | No |
| 2 | BMPR1B | na | 2280 | 0.278 | -0.0017 | No |
| 3 | NOG | na | 5470 | 0.152 | -0.0762 | No |
| 4 | ACVR2B | na | 5771 | 0.143 | -0.0684 | No |
| 5 | ZFYVE16 | na | 6245 | 0.130 | -0.0671 | No |
| 6 | GDF7 | na | 7226 | 0.107 | -0.0831 | No |
| 7 | SMAD6 | na | 7273 | 0.106 | -0.0722 | No |
| 8 | TGFB2 | na | 7601 | 0.100 | -0.0701 | No |
| 9 | CREBBP | na | 7701 | 0.098 | -0.0617 | No |
| 10 | BMPR2 | na | 8199 | 0.088 | -0.0659 | No |
| 11 | BMP5 | na | 8473 | 0.083 | -0.0642 | No |
| 12 | SMAD2 | na | 8821 | 0.078 | -0.0653 | No |
| 13 | AMHR2 | na | 8935 | 0.076 | -0.0598 | No |
| 14 | THBS3 | na | 9127 | 0.073 | -0.0570 | No |
| 15 | BMP8B | na | 9337 | 0.070 | -0.0550 | No |
| 16 | BMP8A | na | 9599 | 0.066 | -0.0550 | No |
| 17 | ROCK1 | na | 9633 | 0.065 | -0.0484 | No |
| 18 | MYC | na | 10444 | 0.054 | -0.0655 | No |
| 19 | SMAD4 | na | 10504 | 0.054 | -0.0610 | No |
| 20 | RPS6KB2 | na | 10512 | 0.054 | -0.0551 | No |
| 21 | SP1 | na | 11109 | 0.046 | -0.0669 | No |
| 22 | NODAL | na | 11819 | 0.038 | -0.0830 | No |
| 23 | SMAD1 | na | 12114 | 0.035 | -0.0875 | No |
| 24 | ID1 | na | 13107 | 0.026 | -0.1131 | No |
| 25 | CDKN2B | na | 13191 | 0.025 | -0.1126 | No |
| 26 | SMAD5 | na | 13995 | 0.019 | -0.1336 | No |
| 27 | RPS6KB1 | na | 14001 | 0.019 | -0.1316 | No |
| 28 | SMAD3 | na | 14170 | 0.017 | -0.1345 | No |
| 29 | RBX1 | na | 14278 | 0.017 | -0.1356 | No |
| 30 | ROCK2 | na | 15225 | 0.009 | -0.1618 | No |
| 31 | THBS4 | na | 15292 | 0.009 | -0.1627 | No |
| 32 | LEFTY1 | na | 15465 | 0.008 | -0.1668 | No |
| 33 | CHRD | na | 15589 | 0.007 | -0.1695 | No |
| 34 | ZFYVE9 | na | 15703 | 0.006 | -0.1721 | No |
| 35 | CUL1 | na | 16128 | 0.003 | -0.1840 | No |
| 36 | BMPR1A | na | 16675 | -0.001 | -0.1997 | No |
| 37 | TFDP1 | na | 17265 | -0.005 | -0.2161 | No |
| 38 | SKP1 | na | 17557 | -0.007 | -0.2237 | No |
| 39 | ACVR2A | na | 18737 | -0.015 | -0.2559 | No |
| 40 | RBL2 | na | 18976 | -0.017 | -0.2609 | No |
| 41 | TGFBR1 | na | 19919 | -0.023 | -0.2854 | No |
| 42 | PPP2R1A | na | 19944 | -0.023 | -0.2834 | No |
| 43 | BMP7 | na | 20858 | -0.030 | -0.3063 | No |
| 44 | RBL1 | na | 21339 | -0.034 | -0.3162 | No |
| 45 | INHBC | na | 21714 | -0.037 | -0.3228 | No |
| 46 | RHOA | na | 21886 | -0.038 | -0.3233 | No |
| 47 | PITX2 | na | 22256 | -0.042 | -0.3291 | No |
| 48 | BMP6 | na | 22607 | -0.046 | -0.3338 | No |
| 49 | MAPK3 | na | 22614 | -0.046 | -0.3287 | No |
| 50 | TNF | na | 22702 | -0.047 | -0.3258 | No |
| 51 | SMURF1 | na | 23663 | -0.058 | -0.3468 | Yes |
| 52 | EP300 | na | 23862 | -0.060 | -0.3456 | Yes |
| 53 | COMP | na | 23987 | -0.062 | -0.3420 | Yes |
| 54 | ID4 | na | 24189 | -0.065 | -0.3404 | Yes |
| 55 | MAPK1 | na | 24626 | -0.071 | -0.3448 | Yes |
| 56 | SMURF2 | na | 24679 | -0.072 | -0.3380 | Yes |
| 57 | LTBP1 | na | 24986 | -0.076 | -0.3381 | Yes |
| 58 | PPP2CA | na | 25060 | -0.077 | -0.3314 | Yes |
| 59 | TGFB1 | na | 25459 | -0.082 | -0.3334 | Yes |
| 60 | THBS1 | na | 25706 | -0.086 | -0.3307 | Yes |
| 61 | ACVR1C | na | 26246 | -0.094 | -0.3354 | Yes |
| 62 | ACVR1 | na | 26478 | -0.098 | -0.3308 | Yes |
| 63 | ID3 | na | 26657 | -0.102 | -0.3242 | Yes |
| 64 | PPP2R1B | na | 26783 | -0.104 | -0.3159 | Yes |
| 65 | INHBB | na | 26936 | -0.107 | -0.3079 | Yes |
| 66 | LEFTY2 | na | 26951 | -0.107 | -0.2960 | Yes |
| 67 | BMP4 | na | 27068 | -0.110 | -0.2868 | Yes |
| 68 | PPP2CB | na | 27315 | -0.115 | -0.2806 | Yes |
| 69 | E2F4 | na | 27415 | -0.117 | -0.2700 | Yes |
| 70 | THBS2 | na | 27689 | -0.123 | -0.2637 | Yes |
| 71 | AMH | na | 27759 | -0.125 | -0.2513 | Yes |
| 72 | E2F5 | na | 28985 | -0.156 | -0.2688 | Yes |
| 73 | GDF6 | na | 29369 | -0.168 | -0.2605 | Yes |
| 74 | SMAD9 | na | 29593 | -0.175 | -0.2468 | Yes |
| 75 | SMAD7 | na | 29924 | -0.185 | -0.2350 | Yes |
| 76 | DCN | na | 30293 | -0.198 | -0.2229 | Yes |
| 77 | BMP2 | na | 30615 | -0.208 | -0.2082 | Yes |
| 78 | ID2 | na | 31675 | -0.247 | -0.2104 | Yes |
| 79 | INHBA | na | 32642 | -0.297 | -0.2041 | Yes |
| 80 | INHBE | na | 32770 | -0.306 | -0.1726 | Yes |
| 81 | GDF5 | na | 33150 | -0.333 | -0.1453 | Yes |
| 82 | TGFB3 | na | 33262 | -0.342 | -0.1091 | Yes |
| 83 | IFNG | na | 33859 | -0.398 | -0.0806 | Yes |
| 84 | FST | na | 34139 | -0.443 | -0.0377 | Yes |
| 85 | ACVRL1 | na | 34306 | -0.475 | 0.0122 | Yes |
Table: GSEA details [plain text format]

  

Fig 2: KEGG\_TGF\_BETA\_SIGNALING\_PATHWAY      
 Blue-Pink O' Gram in the Space of the Analyzed GeneSet

  

Fig 3: KEGG\_TGF\_BETA\_SIGNALING\_PATHWAY: Random ES distribution      
 Gene set null distribution of ES for **KEGG\_TGF\_BETA\_SIGNALING\_PATHWAY**
